# Beyond homogeneity: Assessing the validity of the Michaelis–Menten rate law in spatially heterogeneous environments

**DOI:** 10.1371/journal.pcbi.1012205

**Published:** 2024-06-06

**Authors:** Seolah Shin, Seok Joo Chae, Seunggyu Lee, Jae Kyoung Kim

**Affiliations:** 1 Department of Applied Mathematics, Korea University, Sejong, Republic of Korea; 2 Biomedical Mathematics Group, Pioneer Research Center for Mathematical and Computational Sciences, Institute for Basic Science, Daejeon, Republic of Korea; 3 Department of Mathematical Sciences, KAIST, Daejeon, Republic of Korea; 4 Division of Applied Mathematical Sciences, Korea University, Sejong, Republic of Korea; IFOM - the Firc Insitute of Molecular Oncology, ITALY

## Abstract

The Michaelis–Menten (MM) rate law has been a fundamental tool in describing enzyme-catalyzed reactions for over a century. When substrates and enzymes are homogeneously distributed, the validity of the MM rate law can be easily assessed based on relative concentrations: the substrate is in large excess over the enzyme-substrate complex. However, the applicability of this conventional criterion remains unclear when species exhibit spatial heterogeneity, a prevailing scenario in biological systems. Here, we explore the MM rate law’s applicability under spatial heterogeneity by using partial differential equations. In this study, molecules diffuse very slowly, allowing them to locally reach quasi-steady states. We find that the conventional criterion for the validity of the MM rate law cannot be readily extended to heterogeneous environments solely through spatial averages of molecular concentrations. That is, even when the conventional criterion for the spatial averages is satisfied, the MM rate law fails to capture the enzyme catalytic rate under spatial heterogeneity. In contrast, a slightly modified form of the MM rate law, based on the total quasi-steady state approximation (tQSSA), is accurate. Specifically, the tQSSA-based modified form, but not the original MM rate law, accurately predicts the drug clearance via cytochrome P450 enzymes and the ultrasensitive phosphorylation in heterogeneous environments. Our findings shed light on how to simplify spatiotemporal models for enzyme-catalyzed reactions in the right context, ensuring accurate conclusions and avoiding misinterpretations in *in silico* simulations.

## Author summary

For over a century, scientists have relied on the simple Michaelis–Menten (MM) rate law to explain how enzymes function. The conventional criterion for using the MM rate law has been derived in homogeneous environments where species are evenly dispersed (e.g., *in vitro* experiments). Here, we found that the conventional criterion does not work in intracellular environments where species are heterogeneously distributed and diffuse slowly. However, we find that a slightly modified formula of the MM rate law, based on total quasi-steady state approximation (tQSSA), is accurate even in heterogeneous environments. In particular, this modified formula, unlike the MM rate law, accurately predicts the rate of drug metabolism and ultrasensitive phosphorylation when species are not evenly distributed. Our results provide insight into how to use simplified models for describing enzyme functions in such non-evenly distributed environments.

## Introduction

Enzymes play significant roles in regulating the rates of reactions in living organisms, influencing essential processes such as metabolism, signal transduction, and cellular regulation [1–4]. For over a century, the Michaelis–Menten (MM) equation has been the prevailing framework for describing rates of enzyme-catalyzed reactions [5–11]. Specifically, the MM rate law effectively describes the rate of product (*P*) accumulation in a single enzyme-catalyzed mechanism in terms of substrate concentration (*S*):

$$\begin{array}{c} \frac{dP}{dt}=k_{cat}\frac{E_{T}S}{K_{M}+S}, \end{array}$$ (1)

where *k*_*cat*_ is the catalytic constant, and *K*_*M*_ is the MM constant, and *E*_*T*_ is the total enzyme concentration. The MM rate law was proposed by Henri [5] and Michaelis and Menten [6], and derived by Briggs and Haldane using an approach known as the quasi-steady state approximation (QSSA) [7]. This approximation has been referred to as the standard quasi-steady state approximation (sQSSA) [11–14]. The sQSSA has also been utilized to describe a variety of observable biomolecular interactions between genes and transcription factors [15, 16], and receptors and ligands [17, 18].

The MM rate law based on the sQSSA is accurate when the enzyme concentration is low enough to be *E*_*T*_ ≪ *S*_*T*_ + *K*_*M*_, where *S*_*T*_ is total substrate and product concentration [11–14], referred to as the validity condition of the sQSSA throughout this study. Since the concentrations of enzyme and substrate are roughly comparable in protein interaction reactions, the sQSSA model can be inaccurate [11, 19]. To resolve this inaccuracy of the sQSSA model, an alternative model based on the total quasi-steady state approximation (tQSSA) was proposed [11, 13, 14, 20–23]. Even when the concentration of enzymes is high, and thus the validity condition of the sQSSA is not satisfied, the tQSSA model is accurate [11, 13, 14, 20–23]. Thus, the tQSSA model has been recognized as a more reliable approximation tool for the enzyme–catalyzed reaction model than the sQSSA model.

Both the sQSSA and the tQSSA models are based on an ordinary differential equation (ODE) that assumes homogeneous distributions of species, including enzymes and substrates. However, this assumption appears not to be valid within the cell, unlike in *in vitro* experimental environments. Specifically, enzymes are localized in particular organelles depending on their type and function. For instance, enzymes managing the respiratory chain and oxygen reduction reactions are highly concentrated in mitochondria [24]. Furthermore, cytochrome P450 (CYP) enzymes, essential for drug metabolism, are located in microsomes in the endoplasmic reticulum (ER) of liver cells [25]. Thus, it would be inaccurate to use an ODE to describe enzyme-catalyzed reactions in cells where the assumption of the homogeneous distribution is violated. Instead, employing a partial differential equation (PDE) that accounts for the spatial distribution of chemical species would be a more valid approach.

Previous studies have shown that the applicability of the sQSSA, and thus the MM rate law, can be extended to PDE when the local concentration can be assessed across the entire spatial domain. Specifically, when *E*_*T*_ ≪ *S*_*T*_ + *K*_*M*_ (the validity condition of the sQSSA in ODE) is satisfied at each point within the domain, using the MM rate law in PDE is accurate under various diffusion conditions: (1) when the diffusion time scale is shorter than the fast reaction time scale or longer than the slow reaction time scale [26], (2) when the diffusion time scale aligns with the slow kinetic time scale [27], and (3) when the dissociation of the complex is significantly faster than the diffusion [28]. However, verifying this validity condition necessitates measuring enzyme and substrate concentrations at every point within the entire cell, which is impossible. On the other hand, the spatial average concentrations of enzyme and substrate can be measured using techniques such as western blot [29], UV-Vis spectroscopy [30] and Enzyme-Linked Immunosorbent Assay [31]. This necessitates a validity condition based on spatial average concentrations rather than the local concentration.

Here we investigated whether the sQSSA in PDE is valid when ${\bar{E}}_{T}\ll {\bar{S}}_{T}+K_{M}$, where ${\bar{E}}_{T}$ and ${\bar{S}}_{T}$ are the spatial average of the total enzyme concentration and the total substrate and product concentration across the domain, respectively. Specifically, we showed that the sQSSA in PDE can still be accurate although the spatial averages of molecular concentrations do not satisfy ${\bar{E}}_{T}\ll {\bar{S}}_{T}+K_{M}$, when the substrate and the enzyme are localized in different regions and their diffusion is slower than slow reactions. Conversely, we found that the sQSSA in PDE may introduce substantial errors, even if the spatial average concentrations satisfy ${\bar{E}}_{T}\ll {\bar{S}}_{T}+K_{M}$, especially when the substrate and the enzyme are localized in the same region under conditions of slow diffusion. As a result, employing the sQSSA model to simulate drug metabolism in the liver, where the CYP enzyme is localized in the ER, leads to considerable error in predicting drug clearance, the rate of a drug’s breakdown in the liver. Unlike the sQSSA, using the tQSSA in PDE was accurate for all of these cases because the tQSSA in ODE is generally valid regardless of enzyme concentration. These findings imply that in heterogeneous spaces, where validating the sQSSA at every point is challenging, utilizing the sQSSA in PDE poses a risk. Thus, the tQSSA should be used to model enzyme-catalyzed reactions in heterogeneous spaces.

## Results

### QSSAs for ODE describing enzyme-catalyzed reactions

**Fig 1:**
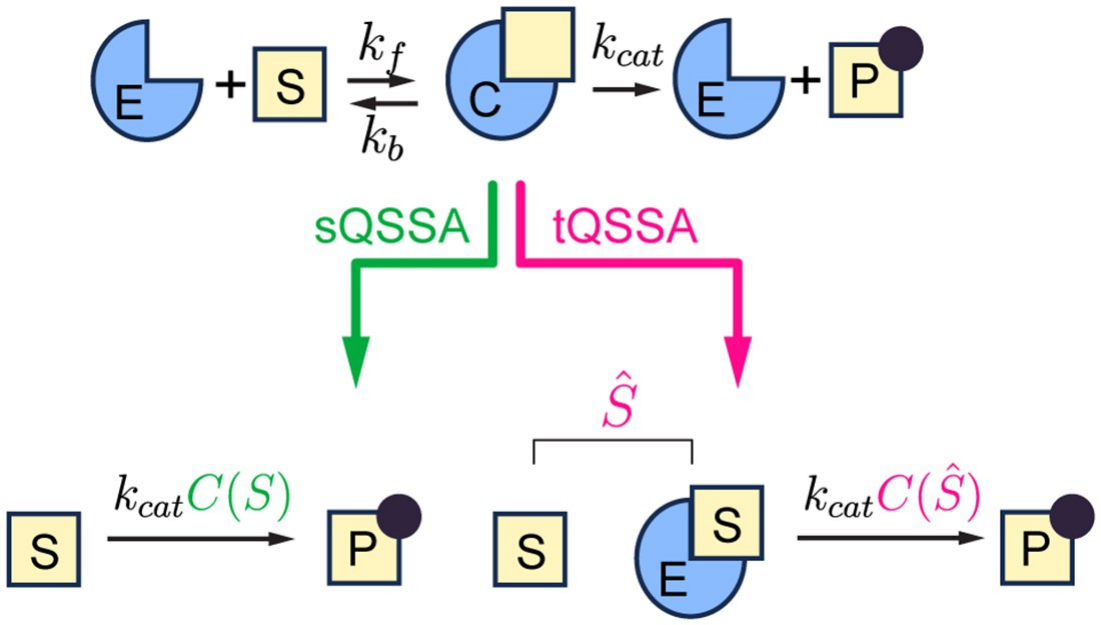
QSSAs for an enzyme-catalyzed reaction. A model describing an enzyme-catalyzed reaction based on mass-action kinetics (Eqs 2 and 9) can be simplified using either the sQSSA_o_ (Eqs 5 and 11) and the sQSSA_p_ (Eq 11) or the tQSSA_o_ (Eq 8) and the tQSSA_p_ (Eq 13). Both approximations of QSS are defined in terms of *S* or $\hat{S}=S+C$.

An enzyme-catalyzed reaction can be described by the following ODE system based on mass-action kinetics (Fig 1):

$$\begin{array}{c} \begin{array}{cc} \frac{dS}{dt} & =-k_{f}S\cdot E+k_{b}C,\\ \frac{dE}{dt} & =-k_{f}S\cdot E+k_{b}C+k_{cat}C,\\ \frac{dC}{dt} & =k_{f}S\cdot E-k_{b}C-k_{cat}C,\\ \frac{dP}{dt} & =k_{cat}C, \end{array} \end{array}$$ (2)

where *E* and *C* are the concentration of the enzyme and the complex, respectively. The enzyme reversibly binds the substrate to form the complex and then the complex irreversibly dissociates into product and enzyme. Note that *E*_*T*_ ≡ *E* + *C* and *S*_*T*_ ≡ *S* + *C* + *P* are always conserved. By utilizing conservation laws, this model can be effectively expressed as an ODE system with two variables, *S* and *C*, as follows:

$$\begin{array}{c} \begin{array}{cc} \frac{dS}{dt} & =-k_{f}S\cdot \left(E_{T}-C\right)+k_{b}C,\\ \frac{dC}{dt} & =k_{f}S\cdot \left(E_{T}-C\right)-k_{b}C-k_{cat}C, \end{array} \end{array}$$ (3)

where *P* can be simply obtained by *P* = *S*_*T*_ − *C* − *S*. We can further simplify the model by assuming that *C* reaches a quasi-steady state (QSS) more rapidly than *S* and then both *C* and *S* slowly track along the QSS. The approximation to QSS (i.e., QSSA) can be obtained by solving *dC*/*dt* = 0:

$$\begin{array}{c} C\left(S\right)=\frac{E_{T}S}{K_{M}+S}, \end{array}$$ (4)

where $K_{M}=\frac{k_{b}+k_{cat}}{k_{f}}$ is the MM constant. By substituting Eq 4 for Eq 3, the MM rate law can be derived:

$$\begin{array}{c} \frac{dP}{dt}=k_{cat}\frac{E_{T}S}{K_{M}+S}. \end{array}$$ (5)

As the MM rate law is derived by using the sQSSA for ODE systems, we refer it to as the sQSSA_o_ model throughout the study. The sQSSA_o_ model is accurate when *E*_*T*_ is sufficiently small (i.e., *E*_*T*_ ≪ *S*_*T*_ + *K*_*M*_) [11–14]. Thus, when *E*_*T*_ and *S*_*T*_ are comparable, such as in protein interaction reactions, using the sQSSA_o_ model can lead to significant errors [11, 19]. This inaccuracy arises from treating *S* as a slow variable, even though *S* is also affected by fast binding and unbinding reactions [11, 32]. In other words, the time scale of *S* is not significantly longer than that of *C*, violating the underlying assumption of the sQSSA.

Such inaccuracy can be resolved by introducing the total substrate concentration $\hat{S}=S+C$. By using $\hat{S}$, Eq 3 can be rewritten as follows:

$$\begin{array}{c} \begin{array}{cc} \frac{d\hat{S}}{dt} & =-k_{cat}C,\\ \frac{dC}{dt} & =k_{f}\left(\hat{S}-C\right)\left(E_{T}-C\right)-k_{b}C-k_{cat}C. \end{array} \end{array}$$ (6)

As $\hat{S}$ is now solely affected by a slow reaction (i.e., $\frac{d\hat{S}}{dt}=-k_{cat}C$), the time scale of $\hat{S}$ is significantly longer than that of *C*. Thus, *C* is more likely to reach QSS before $\hat{S}$ changes appreciably. The QSSA of *C* given $\hat{S}$ can be obtained by solving $\frac{dC}{dt}=0$ in Eq 6:

$$\begin{array}{c} C\left(\hat{S}\right)=\frac{1}{2}[E_{T}+\hat{S}+K_{M}-\sqrt{{\left(E_{T}+\hat{S}+K_{M}\right)}^{2}-4E_{T}\hat{S}}]. \end{array}$$ (7)

Substituting this QSSA for *C* (Eq 7) back into the model (Eq 6) results in the reduced model:

$$\begin{array}{c} \begin{array}{cc} \frac{dP}{dt} & =\frac{1}{2}k_{cat}[E_{T}+\hat{S}+K_{M}-\sqrt{{\left(E_{T}+\hat{S}+K_{M}\right)}^{2}-4E_{T}\hat{S}}], \end{array} \end{array}$$ (8)

where $\hat{S}=S_{T}-P$. As this model is derived with the total quasi-steady state approximation for ODE systems [11, 13, 14, 20–23], we referred it to as the tQSSA_o_ model throughout the study.

It was shown that the tQSSA_o_ model is accurate when the condition $\frac{K}{2S_{T}}\frac{E_{T}+K_{M}+S_{T}}{\sqrt{{\left(E_{T}+K_{M}+S_{T}\right)}^{2}-4E_{T}S_{T}}}\ll 1$ is satisfied, where *K* = *k*_*cat*_/*k*_*f*_. Given that $\frac{K}{2S_{T}}\frac{E_{T}+K_{M}+S_{T}}{\sqrt{{\left(E_{T}+K_{M}+S_{T}\right)}^{2}-4E_{T}S_{T}}}<\frac{1}{4}$ is always satisfied, it has been believed that the tQSSA_o_ model is generally accurate [11, 13, 14, 20–23]. In particular, when the concentration of enzymes is high and thus the sQSSA_o_ model is not valid, the tQSSA_o_ model is accurate [11, 13, 14, 20–23]. Although Eilertsen and Schnell recently identified conditions where even the tQSSA_o_ model becomes inaccurate (i.e., the region outside of $\frac{k_{cat}E_{T}}{k_{f}{\left(K_{M}+E_{T}\right)}^{2}}\ll 1$) [33], this limitation of the tQSSA_o_ is confined to a very small region of the parameter space. In particular, even when *S*_*T*_/*K*_*M*_ ≪ 1 and thus the above conditions are not satisfied [33], it was also shown that the tQSSA_o_ model still performs reasonably well. Thus, the tQSSA_o_ model has been recognized as a reliable approximation tool for the enzyme-catalyzed reaction model [11].

### QSSAs for PDE describing enzyme-catalyzed reactions

So far, we have discussed the application of QSSAs in ODE systems, assuming the even distribution of species in space. To incorporate the spatial distribution of species, we can employ the following PDE system [26–28] defined on a bounded region with a smooth boundary Ω:

$$\begin{array}{cccc} \frac{\partial S}{\partial t} & =D_{S}\Delta S-k_{f}S\cdot E+k_{b}C, & \; & \text{in}\;(0,\;\infty )\times \Omega \\ \frac{\partial E}{\partial t} & =D_{E}\Delta E-k_{f}S\cdot E+k_{b}C+k_{cat}C, & \; & \text{in}\;(0,\;\infty )\times \Omega \\ \frac{\partial C}{\partial t} & =D_{C}\Delta C+k_{f}S\cdot E-k_{b}C-k_{cat}C, & \; & \text{in}\;(0,\;\infty )\times \Omega \\ \frac{\partial P}{\partial t} & =D_{P}\Delta P+k_{cat}C, & \; & \text{in}\;(0,\;\infty )\times \Omega \end{array}$$ (9)

where diffusion coefficients of *S*, *E*, *C*, and *P* are denoted as *D*_*S*_, *D*_*E*_, *D*_*C*_, and *D*_*P*_, respectively. In this study, we mainly used a one-dimensional domain Ω = (0, *L*), where *L* is the length of the domain. Besides, we used zero-Neumann boundary conditions and continuous initial conditions (ICs) *S*(0, *x*) = *S*_0_(*x*), *E*(0, *x*) = *E*_0_(*x*), *C*(0, *x*) = *C*_0_(*x*), and *P*(0, *x*) = *P*_0_(*x*).

Analogous to the ODE system, we can express Eq 9 as follows by using *E*_*T*_ = *E* + *C*:

$$\begin{array}{cccc} \frac{\partial S}{\partial t} & =D_{S}\Delta S-k_{f}S\left(E_{T}-C\right)+k_{b}C, & \; & \text{in}\;(0,\infty )\times \Omega \\ \frac{\partial E_{T}}{\partial t} & =D_{E}\Delta \left(E_{T}-C\right)+D_{C}\Delta C, & \; & \text{in}\;(0,\infty )\times \Omega \\ \frac{\partial C}{\partial t} & =D_{C}\Delta C+k_{f}S\left(E_{T}-C\right)-k_{b}C-k_{cat}C, & \; & \text{in}\;(0,\infty )\times \Omega \\ \frac{\partial P}{\partial t} & =D_{P}\Delta P+k_{cat}C, & \; & \text{in}\;(0,\infty )\times \Omega \end{array}$$ (10)

Note that *E*_*T*_ is no longer pointwise conserved because the diffusion can change the concentration of the enzyme. The above PDE system (Eq 10) can be simplified by applying QSSA as in the ODE system when the time scales are separated. Since species not only react but also diffuse throughout the cell, it is essential to compare the diffusion time scale with the reaction time scale. When the diffusion time scale is comparable to or shorter than the fast reaction time scale, the concentrations of each species are rapidly homogenized, resulting in similar dynamics to ODE at each point [26]. Thus, we focused on the situation when the diffusion time scale is comparable to or longer than the slow reaction time scale. Under this condition, *C* reaches QSS before the concentrations of other species change significantly by diffusion and reaction [27]. Then, by using the sQSSA_o_ ($C\left(S\right)=\frac{E_{T}S}{S+K_{M}}$), the PDE model (Eq 10) can be reduced as follows [27]:

$$\begin{array}{cccc} \frac{\partial S}{\partial t} & =D_{S}\Delta S-k_{cat}C\left(S\right), & \; & \text{in}\;(0,\infty )\times \Omega \\ \frac{\partial E_{T}}{\partial t} & =D_{E}\Delta \left(E_{T}-C\left(S\right)\right)+D_{C}\Delta C\left(S\right), & \; & \text{in}\;(0,\infty )\times \Omega \\ \frac{\partial P}{\partial t} & =D_{P}\Delta P+k_{cat}C\left(S\right), & \; & \text{in}\;(0,\infty )\times \Omega \end{array}$$ (11)

where $C\left(S\right)=\frac{E_{T}S}{S+K_{M}}$. As the sQSSA_o_ is used in the PDE system, we refer to this model (Eq 11) as the sQSSA_p_.

As in the ODE (Eq 6), we introduce a slow variable $\hat{S}=S+C$ to the PDE system (Eq 10) as follows:

$$\begin{array}{cccc} \frac{\partial \hat{S}}{\partial t} & =D_{S}\Delta \left(\hat{S}-C\right)+D_{C}\Delta C-k_{cat}C, & \; & \text{in}\;(0,\infty )\times \Omega \\ \frac{\partial E_{T}}{\partial t} & =D_{E}\Delta \left(E_{T}-C\right)+D_{C}\Delta C, & \; & \text{in}\;(0,\infty )\times \Omega \\ \frac{\partial C}{\partial t} & =D_{C}\Delta C+k_{f}\left(\hat{S}-C\right)\left(E_{T}-C\right)-k_{b}C-k_{cat}C, & \; & \text{in}\;(0,\infty )\times \Omega \\ \frac{\partial P}{\partial t} & =D_{P}\Delta P+k_{cat}C. & \; & \text{in}\;(0,\infty )\times \Omega \end{array}$$ (12)

Then by using the tQSSA_o_ of $C(\hat{S})$ (Eq 7), we obtained the following reduced PDE system:

$$\begin{array}{cccc} \frac{\partial \hat{S}}{\partial t} & =D_{S}\Delta \left(\hat{S}-C\left(\hat{S}\right)\right)+D_{C}\Delta C\left(\hat{S}\right)-k_{cat}C\left(\hat{S}\right), & \; & \text{in}\;(0,\infty )\times \Omega \\ \frac{\partial E_{T}}{\partial t} & =D_{E}\Delta \left(E_{T}-C\left(\hat{S}\right)\right)+D_{C}\Delta C\left(\hat{S}\right), & \; & \text{in}\;(0,\infty )\times \Omega \\ \frac{\partial P}{\partial t} & =D_{P}\Delta P+k_{cat}C\left(\hat{S}\right), & \; & \text{in}\;(0,\infty )\times \Omega \end{array}$$ (13)

where $C\left(\hat{S}\right)=\frac{1}{2}[E_{T}+\hat{S}+K_{M}-\sqrt{{\left(E_{T}+\hat{S}+K_{M}\right)}^{2}-4E_{T}\hat{S}}]$. We refer to this model (Eq 13) as the tQSSA_p_.

### The accuracy of the sQSSA_p_, but not the tQSSA_p_ varies depending on whether the environment is homogeneous or heterogeneous

We explored the accuracy of the sQSSA_p_ (Eq 11) and the tQSSA_p_ (Eq 13) in both homogeneous and heterogeneous environments, by comparing their behaviors to those of the full model (Eq 9). To simulate these models, we first chose biologically realistic values for the parameters (*D*_*S*_, *D*_*E*_, *D*_*C*_, *D*_*P*_, *k*_*f*_, *k*_*b*_, *k*_*cat*_, and *L*). For instance, we assigned a diffusion coefficient of D_*_ = 0.2*μm*^2^/*s* to all species, which is lower than typical protein diffusion coefficients [34] but corresponds to the diffusion coefficient of large-sized proteins (e.g., PER2 protein complex) [35]. Furthermore, since the protein-protein binding occurs with a rate on the order of 10^6^*M*^−1^*s*^−1^, often ranging up to ∼10^8^*M*^−1^*s*^−1^ [36, 37], we have used *k*_*f*_ = 3.4 ⋅ 10^6^*M*^−1^*s*^−1^. We set *k*_*b*_ and *k*_*cat*_ to 60*s*^−1^ and 3.2*s*^−1^, respectively, ensuring that *K*_*M*_, *k*_*cat*_, and *k*_*cat*_/*K*_*M*_ are in the range of typical enzyme kinetic parameters [38]. Finally, the length of the domain Ω, *L*, was set to 30*μm*, which falls within the human cell size range [39].

**Fig 2:**
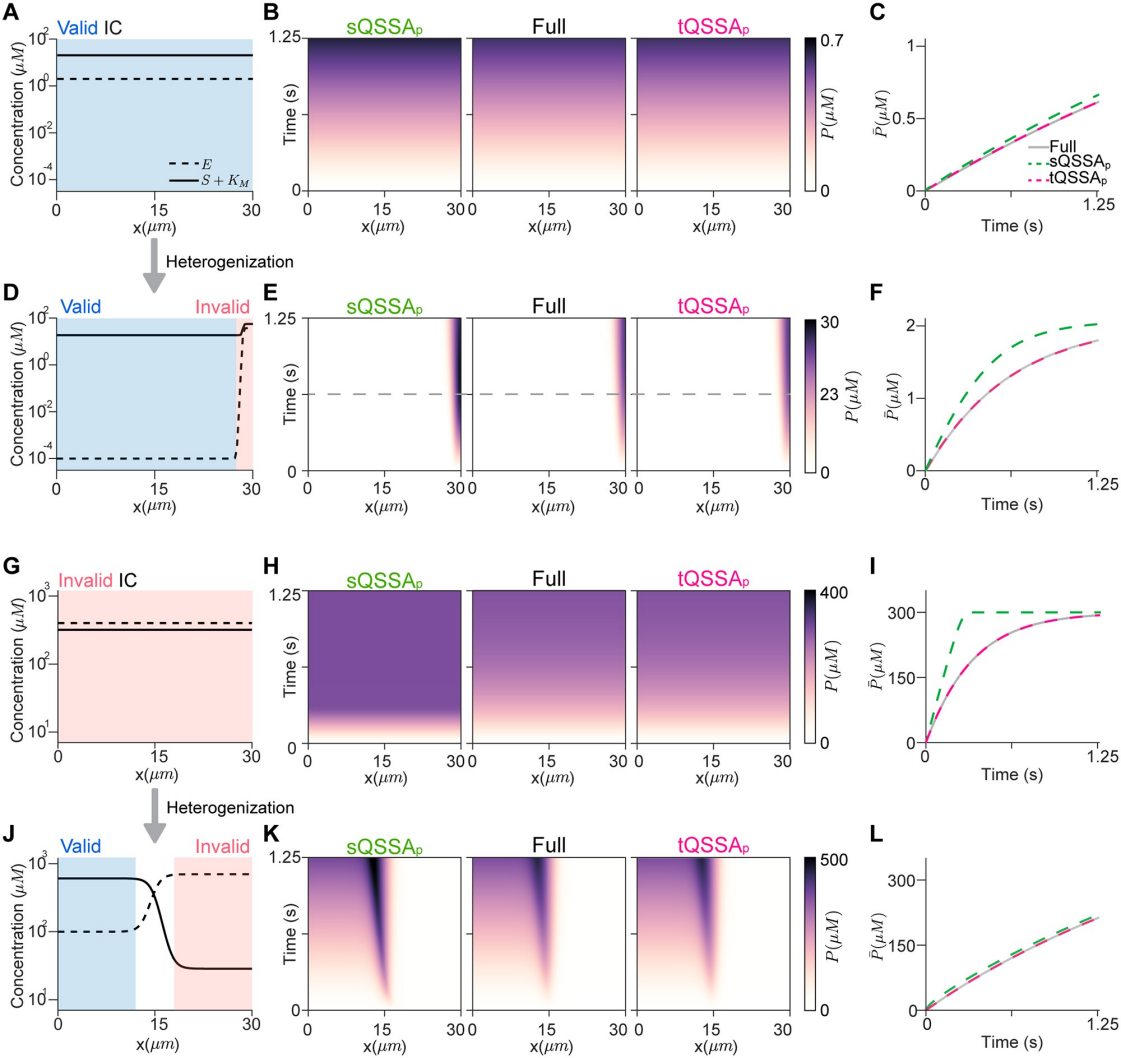
The accuracy of the sQSSA_p_ and the tQSSA_p_ in heterogeneous environments. **(A)** Homogeneous ICs that satisfy the validity condition of the sQSSA_o_ throughout the domain (blue region). *E*_0_ ≡ 2*μM*, *S*_0_ ≡ 2*μM* and *K*_*M*_ = 18.5*μM*. **(B-C)** Both the sQSSA_p_ and the tQSSA_p_ accurately capture the production of *P* throughout the domain (B) and the spatial average $\bar{P}$ (C). **(D)** The ICs in (A) were heterogenized so that the validity condition of the sQSSA_o_ model does not hold near *x* = 30*μm* (red region), unlike the other region (blue region). Here, *E*_0_(*x*) = *S*_0_(*x*) = 18.5(tanh(20*x*/3 − 190) + 1) + 10^−4^*μM*, which maintain the spatial average concentration with the ICs in (A), was used. **(E-F)** The tQSSA_p_ model, but not the sQSSA_p_ model, is accurate. **(G)** The homogeneous ICs that do not satisfy the validity condition of the sQSSA_o_ through the domain. Here, *E*_0_ ≡ 400*μM*, *S*_0_ ≡ 300*μM*, and *K*_*M*_ = 18.5*μM*. **(H-I)** The tQSSA_p_ model, but not the sQSSA_p_ model, is accurate. **(J)** The ICs in (G) were heterogenized so that the validity condition of the sQSSA_o_ is satisfied in *x* < 15*μm* (blue region), but not in *x* ≥ 15*μm* (red region). Here, *E*_0_(*x*) = 300(tanh(2*x*/3 − 10) + 1) + 100*μM* and *S*_0_(*x*) = 290(tanh(10 − 2*x*/3) + 1) + 10*μM*, which keep the spatial average concentration with the ICs in (G), were used. **(K-L)** Both the sQSSA_p_ and tQSSA_p_ models are accurate. For all simulations, *C*_0_ ≡ 0*μM*, *P*_0_ ≡ 0*μM* and *k*_*f*_ = 3.4 ⋅ 10^6^*M*^−1^*s*^−1^, *k*_*b*_ = 60*s*^−1^, *k*_*cat*_ = 3.2*s*^−1^, *D*_*E*_ = *D*_*S*_ = *D*_*C*_ = *D*_*P*_ = D_*_ = 0.2*μm*^2^/*s*.

We first investigated the homogeneous ICs so that the validity condition of the sQSSA_o_ (*E*_*T*_ ≪ *S*_*T*_ + *K*_*M*_) is satisfied at every point of the domain (Fig 2A). In this case, as expected, both the sQSSA_p_ and the tQSSA_p_ accurately capture *P* of the full model at each point (Fig 2B), and thus the spatial average of *P*, $\bar{P}$ (Fig 2C).

Next, we made the ICs heterogeneous while maintaining the spatial average concentrations of Fig 2A, so that the spatial average concentrations satisfy the validity condition of the sQSSA_o_ (${\bar{E}}_{T}\ll {\bar{S}}_{T}+K_{M}$) (Fig 2D). Specifically, we increased the concentrations of *S* and *E* near *x* = 30*μm* and decreased the concentrations in other regions. As a result, the validity condition of the sQSSA_o_ (*S*_*T*_ + *K*_*M*_ ≈ *E*_*T*_) is not satisfied near *x* = 30*μm* (Fig 2D). In this region, the sQSSA_p_ overestimates *P* of the full model (Fig 2E). Since the majority of *S* is localized at this invalid region, the majority of *P* is generated in that region, and thus, the sQSSA_p_ also overestimates $\bar{P}$ compared to the full model (Fig 2F). On the other hand, even with these heterogeneous ICs, the tQSSA_p_ accurately simulates *P* (Fig 2E and 2F).

The impact of a heterogeneous environment on the accuracy of the sQSSA_p_ may diminish with faster diffusion because the initial spatial distribution of species is homogenized and the spatial averages of the concentrations satisfy the validity of the sQSSA_o_ (Fig 2A). Indeed, as the diffusion coefficient (D_*_) increases, the accuracy of the sQSSA_p_ becomes accurate (S1 Fig). Specifically, when the diffusion time scale (*L*^2^/D_*_) is comparable to the time scale of the slowest reaction (1/*k*_*cat*_), the sQSSA_p_ accurately captures the behavior of the full model. On the other hand, when the diffusion time scale is ∼100-fold slower than the time scale of the slowest reaction, the sQSSA_p_ is inaccurate. Taken together, the inaccuracy of the sQSSA_p_ arises when the diffusion is slower than all of the reactions.

We next investigated the opposite case: can the sQSSA_p_ be accurate, even if ${\bar{E}}_{T}\ll {\bar{S}}_{T}+K_{M}$ is not satisfied? For this, we first used the homogeneous ICs where the validity condition of the sQSSA_o_ is not satisfied at any point (Fig 2G). Obviously, the sQSSA_p_ overestimates both *P* and $\bar{P}$ of the full model (Fig 2H and 2I). In contrast, the tQSSA_p_ accurately captures the dynamics of the full model (Fig 2H and 2I).

Now, we heterogenized the ICs while maintaining the spatial average concentrations of Fig 2G (Fig 2J). Specifically, by altering the distribution of *S* and *E*, we made the validity condition of the sQSSA_o_ be satisfied for *x* < 15*μm*, but not for *x* > 15*μm*. With these heterogeneous ICs, unexpectedly, the sQSSA_p_ accurately captures the dynamics of the full model (Fig 2K and 2L). This is because most of *S* is localized where the validity condition of the sQSSA_o_ is satisfied, and thus the majority of *P* is generated in the valid region (Fig 2J, blue region). As a result, the sQSSA_p_ can accurately simulate the production rate of *P*. The tQSSA_p_ also accurately captures the dynamics of the full model (Fig 2K and 2L).

Taken together, the validity of the sQSSA_p_ critically depends on the spatial distribution of the ICs. The sQSSA_p_ can be invalid even when the spatial average concentration satisfies the validity condition of the sQSSA_o_ (${\bar{E}}_{T}\ll {\bar{S}}_{T}+K_{M}$), and conversely, the sQSSA_p_ can be valid even when ${\bar{E}}_{T}\ll {\bar{S}}_{T}+K_{M}$ is not satisfied. Therefore, using the validity condition of the sQSSA_o_ based on spatial average concentrations of species is not enough to determine the validity of the sQSSA_p_. On the other hand, the tQSSA_p_ is accurate regardless of the spatial distribution of the ICs.

### Unlike the tQSSA_p_, the sQSSA_p_ may not be applicable in the *in vivo* environment where the enzyme is localized

**Fig 3:**
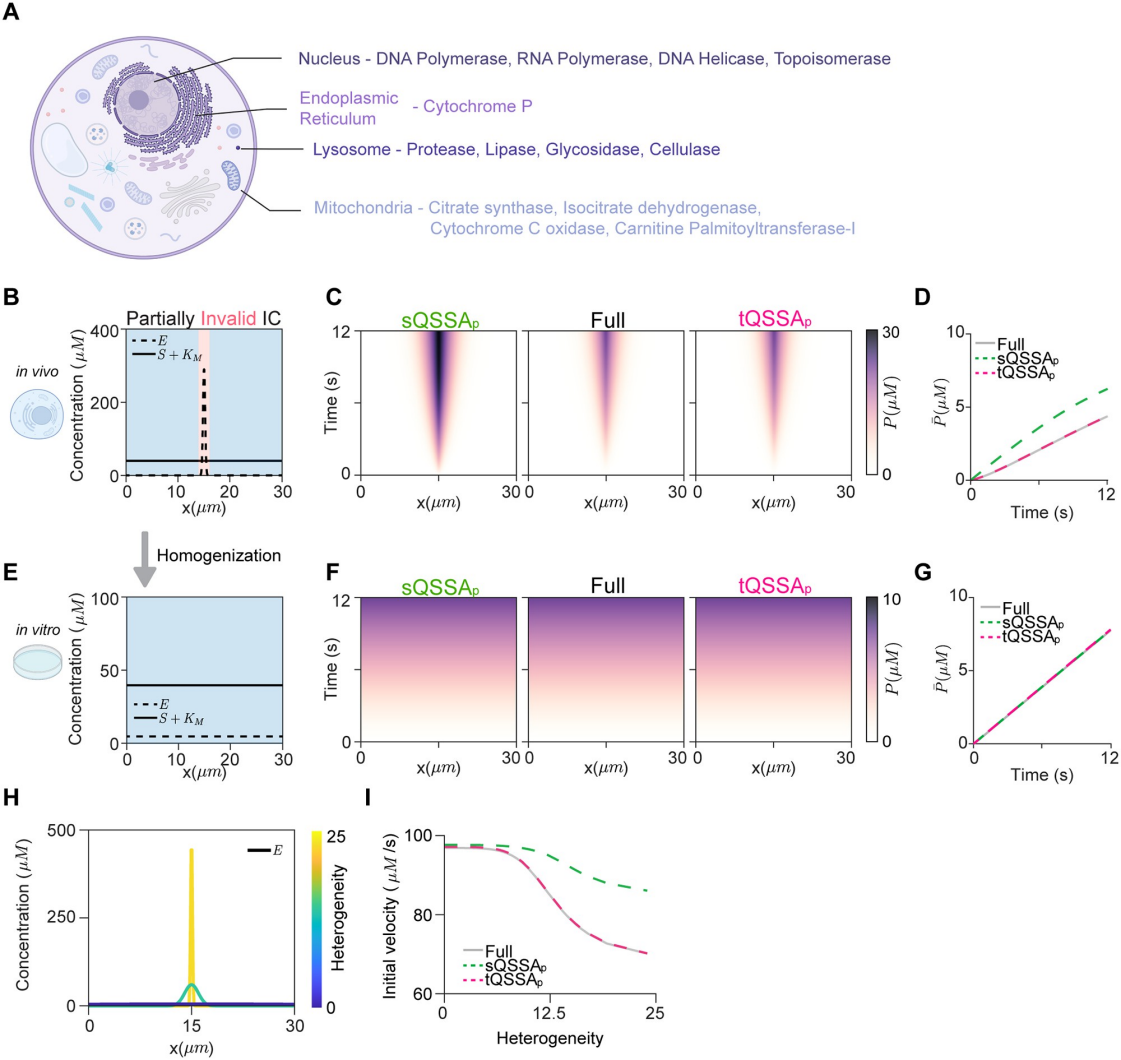
The sQSSA_p_, but not the tQSSA_p_, poses a risk when the enzyme is localized within cells, unlike in an *in vitro* experiment. **(A)** Enzymes localized within cellular organelles. **(B)** The heterogeneous ICs where the sQSSA_o_ are invalid near *x* = 15*μm* (red region) but valid in the other region (blue region). Here *S*_0_ ≡ 39 *μM*, *E*_0_(*x*) = 5 ⋅ *f*(*x*)*μM*, where *f*(*x*) is the normalized probability density function of the normal distribution with the mean of 15 *μm* and the standard deviation of 0.2 *μm*. **(C-D)** Unlike the sQSSA_p_ model, the tQSSA_p_ model accurately captures the production of *P* throughout the domain (C) and its spatial average $\bar{P}$ (D). **(E)** The ICs in (B) were homogenized so that the validity condition of the sQSSA_o_ is satisfied throughout the domain while maintaining the spatial average concentration. Specifically, *S*_0_ ≡ 39 *μM* and *E*_0_ ≡ 5*μM* were used. **(F-G)** Both the sQSSA_p_ and tQSSA_p_ models are accurate. **(H)** Enzyme distributions with varying heterogeneity were constructed using *E*_0_(*x*) = 5 ⋅ *f*(*x*|*σ*) *μM*, where *f*(*x*|*σ*) is the normalized probability density function of the normal distribution with the mean of 15*μm* and the standard deviation of *σ*. As *σ* decreases, the heterogeneity increases (see Method for details). **(I)** For the enzyme distribution with varying heterogeneity, the tQSSA_p_ model, but not the sQSSA_p_ model, accurately captures the initial velocity (I). For all simulations, we used *k*_*f*_ = 6.7 ⋅ 10^5^*M*^−1^*s*^−1^, *k*_*b*_ = 0.53*s*^−1^, *k*_*cat*_ = 0.13*s*^−1^, *K*_*M*_ = 1*μM*, and the diffusion coefficients: *D*_*E*_ = *D*_*C*_ = 0*μm*^2^/*s*, and *D*_*P*_ = *D*_*S*_ = 0.2*μm*^2^/*s*. Some parts of Fig 3 were retrieved from Biorender.

In the previous section, we demonstrated that the validity of the sQSSA_p_ cannot be ensured solely by the spatial average concentrations when species are not homogeneously distributed within a domain. This situation is common within cells, as specific enzymes are localized within distinct subcellular organelles such as the nucleus, the ER, lysosomes, and mitochondria (Fig 3A). For instance, in hepatocytes, cytochrome P450 enzymes, which metabolize drugs, are localized in the ER [25].

To mimic *in vivo* environments with the localized enzyme distribution, we utilized the ICs where the enzyme is localized in a narrow region near *x* = 15*μm* (Fig 3B, dashed line), while the substrate is uniformly distributed throughout the entire cell (Fig 3B, solid line). The ICs do not satisfy the validity condition of the sQSSA_o_ in a narrow region near *x* = 15*μm*. As a result, the sQSSA_p_ model overestimates the production of *P* (Fig 3C and 3D). This is because the majority of the *P* is produced in the region where the enzyme is localized, and thus sQSSA_o_ is not valid. However, the tQSSA_p_ model accurately captures the dynamics of the full model (Fig 3C and 3D). Taken together, it is safer to use the tQSSA_p_ model than the sQSSA_p_ model in an *in vivo* environment.

*In vitro* experiments are usually performed with enzyme and substrate concentrations similar to those in *in vivo* experiments. To mimic this *in vitro* environment, we homogenized the ICs in Fig 3B while maintaining the spatial average concentrations (Fig 3E). With this homogenization, the validity condition of the sQSSA_o_ (*E*_*T*_ ≪ *S*_*T*_ + *K*_*M*_) is satisfied throughout the domain. As a result, not surprisingly, in this case, both the sQSSA_p_ and tQSSA_p_ models accurately approximate the dynamics of the full model (Fig 3F and 3G). This indicates that although the sQSSA_p_ model provides a precise approximation of the full model in *in vitro*, it cannot be directly translated to the *in vivo* environment. This is consistent with previous studies showing that utilizing sQSSA_o_ to extrapolate *in vitro* drug clearance to *in vivo* drug clearance results in a substantial overestimation of the drug clearance, while the tQSSA_o_ model provides a reliable estimate for drug clearance [40, 41].

Furthermore, in *in vivo* environments, directly measuring enzyme concentrations throughout cells (e.g., CYP in hepatocytes) is challenging. In the absence of such information, one can investigate how drug clearance changes depending on the heterogeneity of the enzyme distribution in the cell. As the heterogeneity of the initial distribution of *E* increases (Fig 3H), the initial velocity of production formation considerably decreases (Fig 3I). This is accurately captured by the tQSSA_p_ model, but not the sQSSA_p_ model (Fig 3I). This indicates that the sQSSA_p_ model tends to overestimate drug clearance to a larger extent as the cellular environment becomes more heterogeneous.

### Unlike the tQSSA_p_, the sQSSA_p_ can create artificial ultrasensitivity and spatial patterns

**Fig 4:**
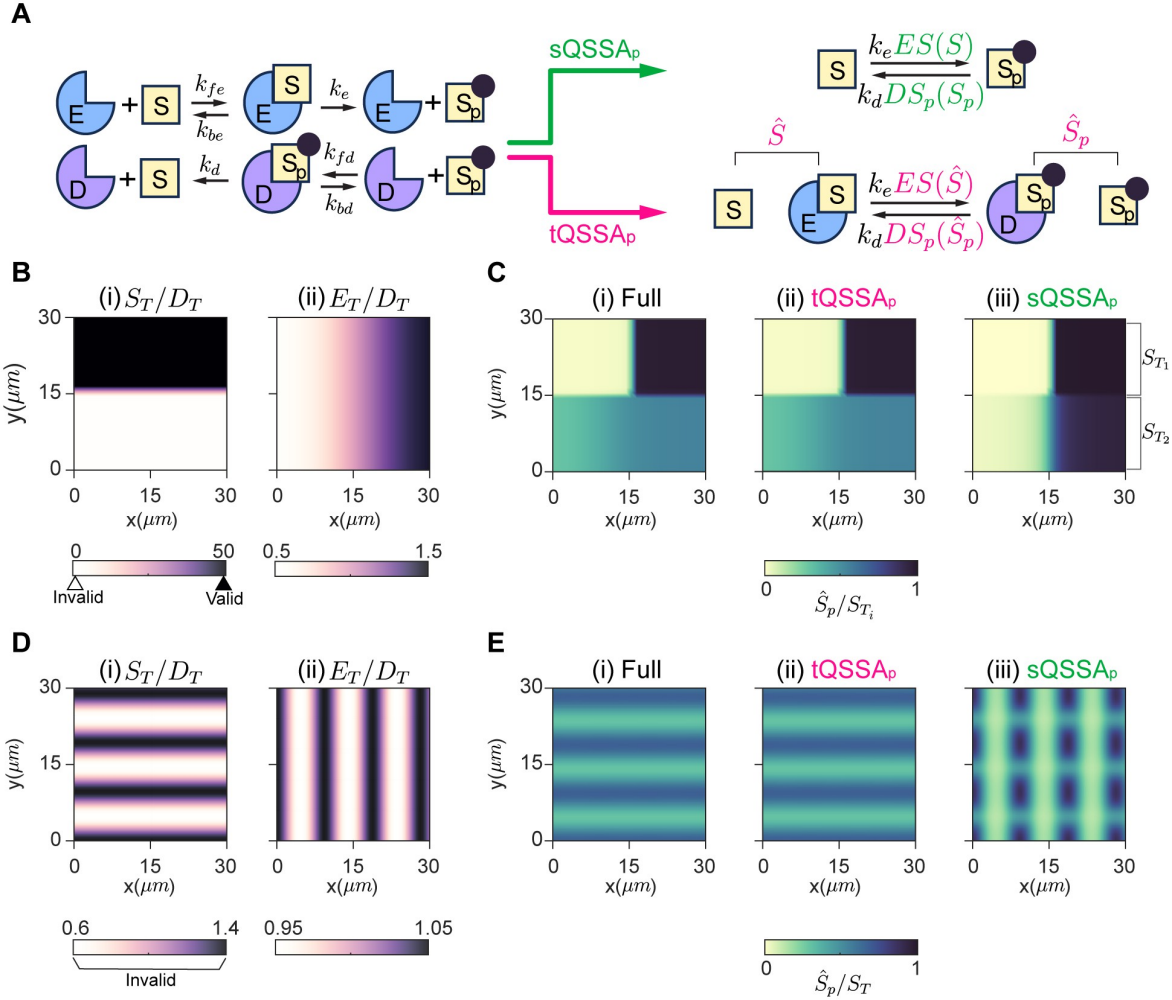
The sQSSA_p_ generates false patterns due to artificial ultrasensitivity. **(A)** The model diagram depicting the GK mechanism. The full model (Eq 14) based on mass-action kinetics can be reduced by replacing *ES* and *DS*_*p*_ with either the sQSSA_p_ (Eq 15) or the tQSSA_p_ (Eq 16). **(B)** The heterogeneous ICs where the sQSSA_o_ is valid in *y* > 15*μm* (black triangle), but invalid in *y* < 15*μm* (white triangle). For this, we used *S*_0_(*x*, *y*) = 500 tanh(3.3*y* − 50) + 520*μM*, *E*_0_(*x*, *y*) = 10 tanh(0.2*x* − 3) + 20*μM*, and *D*_0_ ≡ 20*μM*. **(C)** In *y* > 15*μm*, the ultrasensitivity of the full model (i) is accurately captured by both the tQSSA_p_ (ii) and sQSSA_p_ models (iii). On the other hand, in *y* < 15*μm*, the sQSSA_p_ model generates artificial ultrasensitivity (iii) unlike the full (i) and tQSSA_p_ models (ii). Here, ${\hat{S}}_{p}/S_{T}=S_{p}/S_{T}$ for the sQSSA_p_ model, because *DS*_*p*_ is assumed to be negligible. **(D)** Complex ICs where *S*_*T*_/*D*_*T*_ exhibits horizontal stripes and *E*_*T*_/*D*_*T*_ exhibits vertical stripes. These ICs do not satisfy the validity condition of the sQSSA_o_ throughout the domain. For this, we used *S*_0_(*x*, *y*) = 40 cos(2*y*/3) + 100 *μM*, *E*_0_(*x*, *y*) = 5 cos(2*x*/3) + 100*μM*, and *D*_0_ ≡ 100 *μM*. **(E)** The full model (i) and the tQSSA_p_ model (ii) exhibit horizontally striped patterns. In contrast, the sQSSA_p_ model (iii) results in a grid pattern due to the artificial ultrasensitivity. (C) and (E) were obtained when *t* = 37.5*s*. For initial conditions of other species, *S*_*p*,0_ ≡ 0 *μM*, *ES*_0_ ≡ 0*μM*, and *DS*_*p*,0_ ≡ 0 *μM* were used. In addition, we used *k*_*fe*_ = *k*_*fd*_ = 2.22 ⋅ 10^6^*M*^−1^*s*^−1^, *k*_*be*_ = *k*_*bd*_ = 1.84*s*^−1^, *k*_*e*_ = *k*_*d*_ = 0.38*s*^−1^, *K*_*ME*_ = *K*_*MD*_ = 1*μM*, and $\delta_{S}=\delta_{S_{p}}=\delta_{ES}=\delta_{DS_{p}}=0.2\mu m^{2}/s$.

So far, we have investigated how the accuracy of QSSAs can vary in a simple enzyme-catalyzed model. Here, we examined biological systems that encompass multiple enzymes, which can exhibit complex behaviors, such as ultrasensitivity, bistability, and oscillation [42]. One prominent example of such reactions is the Goldbeter–Koshland (GK) mechanism (Fig 4A). The GK mechanism describes two interlocked enzyme-catalyzed reactions with a single substrate [11, 43]. Specifically, the substrate (*S*) is phosphorylated by the kinase (*E*), and the phosphorylated substrate (*S*_*P*_) is dephosphorylated by the phosphatase (*D*). We extended the GK mechanism to the PDE system to represent the spatial distribution of species within the cell in a two-dimensional domain (Ω = (0, *L*) × (0, *L*)) and derived its sQSSA_p_ and tQSSA_p_ models (Fig 4A) (see Methods for details).

We explored whether the sQSSA_p_ and the tQSSA_p_ models capture the behaviors of the full model across varying concentrations of substrate and enzymes. Specifically, we divided the domain into two regions and gave them two different ICs: one with a high *S*_*T*_/*D*_*T*_ ratio (*y* ≥ 15*μm*), and another with a small *S*_*T*_/*D*_*T*_ ratio (*y* < 15*μm*) (Fig 4B(i)). As a result, the ICs satisfy the validity condition of the sQSSA_o_ in *y* ≥ 15*μm* (i.e., $\frac{D_{T}}{K_{MD}+S_{T}}\approx 0.02\ll 1$), but not in *y* < 15*μm* ($\frac{D_{T}}{K_{MD}+S_{T}}\approx 1$).

In *y* ≥ 15*μm*, as *E*_*T*_/*D*_*T*_ increases in a horizontal direction (Fig 4B(ii)), the full model exhibits zero-order ultrasensitivity of phosphorylation at the steady state (Fig 4C(i)). That is, at *E*_*T*_/*D*_*T*_ < 1 (*x* < 15*μm*), the majority of *S* remains unphosphorylated, and thus the steady-state fraction of total phosphorylated substrate (${\hat{S}}_{p}/S_{T}$) is close to zero (Fig 4C(i)). On the other hand, when *E*_*T*_/*D*_*T*_ surpasses 1 at *x* = 15*μm*, the phosphorylation of *S* becomes suddenly predominant, causing ${\hat{S}}_{p}/S_{T}$ to sharply increase and approach 1 (Fig 4C(i)). This ultrasensitivity is successfully captured by both the tQSSA_p_ (Fig 4C(ii)) and the sQSSA_p_ models (Fig 4C(iii)).

In *y* < 15*μm*, the full model does not exhibit ultrasensitivity. That is, ${\hat{S}}_{p}/S_{T}$ gradually increases as *E*_*T*_/*D*_*T*_ increases and becomes saturated near *x* = 15*μm*, where *E*_*T*_/*D*_*T*_ ≈ 1 (Fig 4C(i)). Thus, ${\hat{S}}_{p}/S_{T}$ does not sensitively change around *E*_*T*_/*D*_*T*_ ≈ 1. The tQSSA_p_ accurately captures the smooth changes in phosphorylation of the full model (Fig 4C(ii)). However, the sQSSA_p_ exhibits an ‘artificial’ ultrasensitivity (Fig 4C(iii)). That is, using the sQSSA_p_ to describe the GK mechanism can result in an ‘artificial’ ultrasensitivity.

Ultrasensitivity is important for pattern formation across various biological and chemical reactions such as cell division [42, 44–46]. We examined how this ‘artificial’ ultrasensitivity caused by the sQSSA_p_ affects pattern formation. For this, we used ICs that do not satisfy the validity condition of the sQSSA_o_ across all regions (i.e., $\frac{D_{T}}{K_{MD}+S_{T}}\approx 1$) (Fig 4D(i)). Furthermore, we made ICs of *S*_*T*_/*D*_*T*_ and *E*_*T*_/*D*_*T*_ that have horizontal and vertical striped patterns, respectively (Fig 4D(i) and 4D(ii)). Because *E*_*T*_/*D*_*T*_ changes slightly around one along the *x*-direction, the full model simulates minimal change of ${\hat{S}}_{p}/S_{T}$ along the *x*-direction (Fig 4E(i)). On the other hand, since *S*_*T*_/*D*_*T*_ changes considerably along the *y*-direction, ${\hat{S}}_{p}/S_{T}$ changes considerably along the *y*-direction. As a result, the full model exhibits a horizontally striped pattern (Fig 4E(i)). This is accurately captured by the tQSSA_p_ model (Fig 4E(ii)). However, the sQSSA_p_ model exhibits an artificial grid pattern (Fig 4E(iii)). This artifact arises due to the artificial ultrasensitivity inherent to the sQSSA_p_. In other words, even slight fluctuations in *E*_*T*_/*D*_*T*_ around one cause significant changes in ${\hat{S}}_{p}/S_{T}$ due to this artificial ultrasensitivity (Fig 4C(iii)). Consequently, a vertical striped pattern emerges and combines with the horizontal pattern, resulting in the observed grid-like pattern (Fig 4E(iii)). This indicates that using the MM equation can generate misleading artificial ultrasensitivity and patterns, although it has been frequently utilized to depict pattern formation [47, 48]. Thus, utilizing the tQSSA is a safer option to investigate pattern formation.

## Discussion

Although the MM rate law has been the prevalent means for describing enzyme-catalyzed reactions for over a century [5–11], the applicability of the MM rate law remains elusive when species are heterogeneously distributed. However, our study demonstrated that the validity condition of the sQSSA_p_ (i.e., the MM rate law in a spatiotemporal system) is different from that of the sQSSA_o_ (Fig 2). Specifically, even if the spatial average concentrations of species satisfy the validity condition of the sQSSA_o_, the sQSSA_p_ model can be inaccurate. And conversely, the sQSSA_p_ model can be accurate even if the spatial average concentrations do not satisfy the validity condition of the sQSSA_o_. Thus, the validity condition based on spatial average concentration does not ensure the accuracy of the sQSSA_p_ model. Therefore, to assure its accuracy, it is necessary to verify that ICs satisfy the validity condition of the sQSSA_o_ at each point in the domain. However, this is not feasible because it is challenging to measure concentrations at every point in the cell. Thus, since the tQSSA_p_ is valid in most cases, our findings suggest that the tQSSA_p_ offers the best way to reduce and investigate spatiotemporal models.

For systems with fast diffusion, the dynamics of PDE and ODE models are very similar because the concentrations of each species homogenize quickly [26]. In such systems, the validity condition of the sQSSA_p_ would be similar to that of the sQSSA_o_. However, we found that for systems with slow diffusion, the validity of the sQSSA_p_ becomes different from the sQSSA_o_ (Figs 2 and 4). This indicates that in models involving slow diffusion, such as 1) measuring dopamine concentration in the brain [49] and 2) detecting current differences with biosensors [50–52], the MM rate law could be inaccurate even when the validity condition of the sQSSA_o_ is satisfied. Thus, when reducing spatiotemporal models with slow diffusion, it is safer to use the tQSSA_p_ than the sQSSA_o_ to avoid potential inaccuracies.

Drugs administered into the body are primarily broken down by CYP enzymes located in liver cells [25]. Drug clearance serves as a key pharmacokinetic parameter for predicting human systemic drug disposition, as cited in over 65,000 papers [40, 42, 53–55]. While the MM rate law has conventionally been used to estimate drug clearance, its validity in drug clearance has only recently been verified [40, 41]. Recent studies suggested that this approach is only valid when the spatial average CYP concentration is significantly smaller than the MM constant of the drug (i.e., ${\bar{E}}_{T}/K_{M}<0.1$) [40, 41]. Otherwise, the tQSSA model should be used to predict the drug clearance [40, 41]. However, our findings indicate that this criterion for using the MM rate law, based on the spatial average concentration, can be misleading because the liver cell is heterogeneous (Fig 3). Therefore, even when ${\bar{E}}_{T}/K_{M}<0.1$ is satisfied, utilizing the tQSSA_p_ may be a safer approach for predicting drug clearance.

In experimental settings including *in vivo* environments, accurately measuring concentrations of substances within cells poses a challenge, which hampers the investigation of how spatial differences affect enzyme-catalyzed reactions. While utilizing various initial conditions can represent different levels of heterogeneity, simulating the full model, encompassing all binding and unbinding interactions, is computationally expensive. Therefore, employing simplified models using sQSSA_p_ or tQSSA_p_ is more efficient. However, the sQSSA_p_ model may not accurately reflect the behavior of the full model in the presence of spatial heterogeneity. For example, our simulation showed that the sQSSA_p_ model underestimated the impact of spatial heterogeneity on the initial velocity of enzyme-catalyzed reactions (Fig 3H and 3I). Thus, using the tQSSA_p_ model, which accurately captures the behavior of the full model regardless of spatial heterogeneity, is a reasonable solution. Indeed, the tQSSA_p_ model accurately captures the range of the initial velocities of enzyme-catalyzed reactions depending on the spatial heterogeneity of enzyme distributions (Fig 3I).

We have investigated whether QSSAs in the PDE system provide accurate approximations when species are heterogeneously distributed (Figs 2–4). Our work shows that the accuracy of the tQSSA_p_ model, but not the sQSSA_p_ model, is not affected by the heterogeneity level. However, to determine whether this result is valid universally, it would be essential to investigate this with a broader range of conditions. Furthermore, our findings are based on numerical simulations rather than theoretical derivations. Notably, recent theoretical studies have identified a small region where the tQSSA_o_ becomes inaccurate, although the tQSSA_o_ has been believed to be generally accurate [33, 56]. Therefore, the theoretical investigation of the validity of the tQSSA_p_ should be a crucial focus for future research. Furthermore, a recent study proposed a more accurate model than the MM rate law by using the effective time-delay scheme [57]. Investigating this new scheme in heterogeneous environments would be also interesting in future work.

Even when the tQSSA_p_ accurately approximates the original PDE systems, the tQSSA_p_ may not be accurate in a stochastic context. Using the tQSSA_o_ for stochastic simulations is usually accurate [11, 58–64] and thus has been widely used [65–68]. However, a recent study has shown that using the tQSSA_o_ for stochastic simulations can be inaccurate even though it is accurate in a deterministic simulation in which the molar ratio of *E* and *S* is close to 1:1 and their binding is tight [69]. This implies that the validity condition of the tQSSA_o_ in stochastic systems is different from that in deterministic systems. Therefore, it would be interesting in future work to investigate the validity of the tQSSA_p_ in deterministic and stochastic contexts.

## Materials and methods

### Numerical simulation for a simple enzyme-catalyzed reaction model (1-D model)

In Figs 2 and 3, to simulate the enzyme-catalyzed reaction, we numerically solved PDE systems (Eqs 9, 11 and 13) in a one-dimensional space Ω = (0, *L*). Let *x*_*j*_ = *j*Δ*x* = *jL*/(*N*_*x*_ − 1) be the *N*_*x*_-equispaced grids in Ω, which are referred to as the Fourier collocation points, to implement the cosine-based spectral method. The time step Δ*t* was defined as Δ*t* = *T*/*N*_*t*_, where *T* is the final time and *N*_*t*_ is the total number of time steps. We denote the numerical approximation of *S*(*n*Δ*t*, *x*), *E*(*n*Δ*t*, *x*), *C*(*n*Δ*t*, *x*), *P*(*n*Δ*t*, *x*) by *S*^*n*^, *E*^*n*^, *C*^*n*^, *P*^*n*^, respectively.

To facilitate simulation, we split PDE systems into diffusion and reaction parts using the operator splitting method [70]. That is, for each time step of the simulation, the diffusion and reaction were calculated separately. We first calculated the diffusion parts in Eqs 9, 11, and 13 as follows:

$$\begin{array}{c} \frac{S^{*}-S^{n}}{\Delta t}=D_{S}\Delta S^{*},\\ \frac{E^{*}-E^{n}}{\Delta t}=D_{E}\Delta E^{*},\\ \frac{C^{*}-C^{n}}{\Delta t}=D_{C}\Delta C^{*},\\ \frac{P^{*}-P^{n}}{\Delta t}=D_{P}\Delta P^{*}. \end{array}$$

After obtaining S*, E*, C*, and P* by calculating the diffusion part, the reaction parts were calculated to obtain *S*^*n*+1^, *E*^*n*+1^, *C*^*n*+1^, and *P*^*n*+1^. Specifically, for the full model (Eq 9), the following reaction equations were used.

$$\begin{array}{cc} \frac{S^{n+1}-S^{*}}{\Delta t} & =-k_{f}E^{*}S^{*}+k_{b}C^{*},\\ \frac{E^{n+1}-E^{*}}{\Delta t} & =-k_{f}E^{*}S^{*}+k_{b}C^{*}+k_{cat}C^{*},\\ \frac{C^{n+1}-C^{*}}{\Delta t} & =k_{f}E^{*}S^{*}-k_{b}C^{*}-k_{cat}C^{*},\\ \frac{P^{n+1}-P^{*}}{\Delta t} & =k_{cat}C^{*}. \end{array}$$

For the sQSSA_p_ model (Eq 11), we defined $E_{T}^{*}=E^{*}+C^{*}$ and $C^{*}\left(S^{*}\right)=\frac{E_{T}^{*}S^{*}}{S^{*}+K_{M}}$ to calculate the reaction parts. Then, we obtained *S*^*n*+1^ and *P*^*n*+1^ as follows:

$$\begin{array}{cc} \frac{S^{n+1}-S^{*}}{\Delta t} & =-k_{cat}C^{*}\left(S^{*}\right),\\ \frac{P^{n+1}-P^{*}}{\Delta t} & =k_{cat}C^{*}\left(S^{*}\right). \end{array}$$

Furthermore, using *S*^*n*+1^ and *P*^*n*+1^, we obtained $C^{n+1}=\frac{E_{T}^{*}S^{n+1}}{\left(S^{n+1}+K_{M}\right)}$, and $E^{n+1}=E_{T}^{*}-C^{n+1}$.

For the tQSSA_p_ model (Eq 13), we defined $E_{T}^{*}=E^{*}+C^{*}$, ${\hat{S}}^{*}=S^{*}+C^{*}$ and $C^{*}\left({\hat{S}}^{*}\right)=\frac{1}{2}[E_{T}^{*}+{\hat{S}}^{*}+K_{M}-\sqrt{{\left(E_{T}^{*}+{\hat{S}}^{*}+K_{M}\right)}^{2}-4E_{T}^{*}{\hat{S}}^{*}}]$ to calculate the reaction part. Then the following reaction equations were used to obtain ${\hat{S}}^{n+1}$ and *P*^*n*+1^.

$$\begin{array}{cc} \frac{{\hat{S}}^{n+1}-{\hat{S}}^{*}}{\Delta t} & =-k_{cat}C^{*}\left({\hat{S}}^{*}\right),\\ \frac{P^{n+1}-P^{*}}{\Delta t} & =k_{cat}C^{*}\left({\hat{S}}^{*}\right). \end{array}$$

Then, using ${\hat{S}}^{n+1}$ and *P*^*n*+1^, we obtained $C^{n+1}=\frac{1}{2}[E_{T}^{*}+{\hat{S}}^{n+1}+K_{M}-\sqrt{{\left(E_{T}^{*}+{\hat{S}}^{n+1}+K_{M}\right)}^{2}-4E_{T}^{*}{\hat{S}}^{n+1}}]$, $E^{n+1}=E_{T}^{*}-C^{n+1}$, and $S^{n+1}={\hat{S}}^{n+1}-C^{n+1}$ for the next time step.

For Fig 2, we simulated with *k*_*f*_ = 3.4*μM*^−1^*s*^−1^, *k*_*b*_ = 60*s*^−1^, and *k*_*cat*_ = 3.2*s*^−1^; thus, the MM constant *K*_*M*_ = 18.5*μM*. Furthermore, we used *L* = 30*μm*, *T* = 1.25*s*, *N*_*x*_ = 100, *N*_*t*_ = 2730, resulting in Δ*x* = 0.3*μm* and Δ*t* = 4.6 ⋅ 10^−4^*s*. Diffusion coefficients of *D*_*E*_ = *D*_*S*_ = *D*_*C*_ = *D*_*P*_ = 0.2*μm*^2^/*s* were used. For Fig 3, we simulated with *k*_*f*_ = 0.67*μM*^−1^*s*^−1^, *k*_*b*_ = 0.53*s*^−1^, *k*_*cat*_ = 0.13*s*^−1^, *K*_*M*_ = 1*μM*. Moreover, we used the parameter *L* = 30*μm*, *T* = 12*s*, *N*_*x*_ = 300, *N*_*t*_ = 11921, resulting in Δ*x* = 0.1*μm*, Δ*t* = 0.001*s*, and assumed that the substrate spread quickly *D*_*P*_ = *D*_*S*_ = 0.2*μm*^2^/*s*, *D*_*E*_ = *D*_*C*_ = 0*μm*^2^/*s*.

For Fig 3H and 3I, we utilized various spatial distributions of enzymes. Specifically, the enzyme concentration was described by the normalized probability density function of the normal distribution with the mean of 15*μm* and the standard deviation of *σ*: *E*_0_(*x*) = 5*f*(*x*|*σ*)(*μM*), where $f\left(x|\sigma \right)=\frac{f_{0}\left(x|\sigma \right)}{\int_{0}^{30}f_{0}\left(x|\sigma \right)dx}$ and $f_{0}\left(x|\sigma \right)=\frac{1}{\sigma \sqrt{2\pi }}\text{exp}(-\frac{{\left(x-15\right)}^{2}}{2\sigma^{2}})$. Then, the heterogeneity of the *E*_0_ was defined as follows:

$$\begin{array}{cc} \text{hetrogeneity} & =10\text{log}(\frac{\sigma_{\text{max}}}{\sigma }), \end{array}$$

where *σ* is the standard deviation of the normal distribution, and we set $\sigma_{\text{max}}=25\mu m$ since *E* becomes almost constant across the space when *σ* ≥ 25*μm*.

Additionally, we measured the initial velocity of the enzyme-catalyzed reaction as the average reaction velocity over the first 12 seconds from the initial state, by using: $\frac{\bar{P}\left(t=12s\right)}{12s}$.

### PDE model describing the GK mechanism

In Fig 4, we extended the GK mechanism [11, 43], which consists of two interconnected, single-substrate, enzyme-catalyzed reactions, to the PDE model as follows:

$$\begin{array}{c} \begin{array}{cc} \frac{\partial S}{\partial t} & =\delta_{S}\Delta S-k_{fe}S\cdot E+k_{be}ES+k_{d}DS_{p},\\ \frac{\partial S_{p}}{\partial t} & =\delta_{S_{p}}\Delta S_{p}-k_{fd}S_{p}\cdot D+k_{bd}DS_{p}+k_{e}ES,\\ \frac{\partial E}{\partial t} & =-k_{fe}S\cdot E+k_{be}ES+k_{e}ES,\\ \frac{\partial D}{\partial t} & =-k_{fd}S_{p}\cdot D+k_{bd}DS_{p}+k_{d}DS_{p},\\ \frac{\partial ES}{\partial t} & =\delta_{ES}\Delta ES+k_{fe}S\cdot E-k_{be}ES-k_{e}ES,\\ \frac{\partial DS_{p}}{\partial t} & =\delta_{DS_{p}}\Delta DS_{p}+k_{fd}S_{p}\cdot D-k_{bd}DS_{p}-k_{d}DS_{p}, \end{array} \end{array}$$ (14)

where *δ*_*S*_, $\delta_{S_{p}}$, *δ*_*ES*_, and $\delta_{DS_{p}}$ denote the diffusion coefficients of *S*, *S*_*p*_, *ES*, and *DS*_*p*_, respectively. We assumed that the enzymes (*D* and *E*) do not diffuse. Furthermore, similar to the simple enzyme-catalyzed reaction (Eq 9), zero-Neumann boundary conditions and ICs were used with *S*(0, *x*, *y*) = *S*_0_(*x*, *y*), *S*_*p*_(0, *x*, *y*) = *S*_*p*,0_(*x*, *y*), *E*(0, *x*, *y*) = *E*_0_(*x*, *y*), *D*(0, *x*, *y*) = *D*_0_(*x*, *y*), *ES*(0, *x*, *y*) = *ES*_0_(*x*, *y*), and *DS*_*p*_(0, *x*, *y*) = *DS*_*p*,0_(*x*, *y*).

In this model, *S*_*T*_ = *S* + *S*_*p*_ + *ES* + *DS*_*p*_, *E*_*T*_ = *E* + *ES*, and *D*_*T*_ = *D* + *DS*_*p*_ are concentrations of total substrate and product, total kinase, and total phosphatase, respectively. This full model (Eq 14) can also be reduced by applying the sQSSA_p_, as done in the simple enzyme model (Fig 4A). In particular, *ES* and *DS*_*p*_ are assumed to reach each QSS, resulting in QSSA of $ES\left(S\right)=\frac{E_{T}S}{S+K_{ME}}$, and $DS_{p}\left(S_{p}\right)=\frac{D_{T}S_{p}}{S_{p}+K_{MD}}$, where *K*_*ME*_ = (*k*_*be*_ + *k*_*e*_)/*k*_*fe*_, *K*_*MD*_ = (*k*_*bd*_ + *k*_*d*_)/*k*_*fd*_. This results in the rates of phosphorylation and dephosphorylation in terms of *E*_*T*_, *D*_*T*_, *S*, and *S*_*p*_:

$$\begin{array}{c} \begin{array}{cc} \frac{\partial S}{\partial t} & =\delta_{S}\Delta S-k_{e}ES\left(S\right)+k_{d}DS_{p}\left(S_{p}\right),\\ \frac{\partial S_{p}}{\partial t} & =\delta_{S_{p}}\Delta S_{p}+k_{e}ES\left(S\right)-k_{d}DS_{p}\left(S_{p}\right),\\ \frac{\partial E_{T}}{\partial t} & =\delta_{ES}\Delta ES\left(S\right),\\ \frac{\partial D_{T}}{\partial t} & =\delta_{DS_{p}}\Delta DS_{p}\left(S_{p}\right). \end{array} \end{array}$$ (15)

Alternatively, we introduce $\hat{S}=S+ES$ and ${\hat{S}}_{p}=S_{p}+DS_{p}$ and apply the tQSSA_p_ by substituting *ES* and *DS*_*p*_ with $ES\left(\hat{S}\right)=\frac{1}{2}[E_{T}+\hat{S}+K_{ME}-\sqrt{{\left(E_{T}+\hat{S}+K_{ME}\right)}^{2}-4E_{T}\hat{S}}]$ and $DS_{p}\left({\hat{S}}_{p}\right)=\frac{1}{2}[D_{T}+{\hat{S}}_{p}+K_{MD}-\sqrt{{\left(D_{T}+{\hat{S}}_{p}+K_{MD}\right)}^{2}-4D_{T}{\hat{S}}_{p}}]$, respectively. Then we obtain the following reduced model of the GK mechanism:

$$\begin{array}{c} \begin{array}{cc} \frac{\partial \hat{S}}{\partial t} & =\delta_{S}\Delta \left(\hat{S}-ES\left(\hat{S}\right)\right)+\delta_{ES}\Delta \left(ES\left(\hat{S}\right)\right)-k_{e}ES\left(\hat{S}\right)+k_{d}DS_{p}\left({\hat{S}}_{p}\right)\\ \frac{\partial {\hat{S}}_{p}}{\partial t} & =\delta_{S_{p}}\Delta \left({\hat{S}}_{p}-DS_{p}\left({\hat{S}}_{p}\right)\right)+\delta_{DS_{p}}\Delta \left(DS_{p}\left({\hat{S}}_{p}\right)\right)+k_{e}ES\left(\hat{S}\right)-k_{d}DS_{p}\left({\hat{S}}_{p}\right),\\ \frac{\partial E_{T}}{\partial t} & =\delta_{ES}\Delta ES\left(\hat{S}\right),\\ \frac{\partial D_{T}}{\partial t} & =\delta_{DS_{p}}\Delta DS_{p}\left({\hat{S}}_{p}\right). \end{array} \end{array}$$ (16)

### Numerical simulation for the model describing the GK mechanism (2-D model)

In Fig 4, we simulated a two-dimensional multi-enzyme model. Similar to the 1-D model, we discretized a spatial variable in a two-dimensional spatial domain Ω = (0, *L*) × (0, *L*) by using the cosine-based spectral method with a uniform mesh size Δ*x* = *L*/(*N*_*x*_ − 1), Δ*y* = *L*/(*N*_*y*_ − 1), where $N_{x},N_{y}\in N$. Let the numerical approximation of *S*^*n*^ = *S*(*n*Δ*t*, *x*, *y*), ${S_{p}}^{n}=S_{p}\left(n\Delta t,x,y\right)$, *E*^*n*^ = *E*(*n*Δ*t*, *x*, *y*), *D*^*n*^ = *D*(*n*Δ*t*, *x*, *y*), *ES*^*n*^ = *ES*(*n*Δ*t*, *x*, *y*), and ${DS_{p}}^{n}=DS_{p}\left(n\Delta t,x,y\right)$.

Similar to Figs 2 and 3, we utilized the operator splitting method and spectral method. Therefore, the diffusion and reaction were still calculated separately for each time step. We first calculated the diffusion parts in Eqs 14–16 as follows:

$$\begin{array}{cc} \frac{S^{*}-S^{n}}{\Delta t} & =\delta_{S}\Delta S^{*},\\ \frac{S_{p}^{*}-S_{p}^{n}}{\Delta t} & =\delta_{S_{p}}\Delta S_{p}^{*},\\ \frac{ES^{*}-ES^{n}}{\Delta t} & =\delta_{ES}\Delta ES^{*},\\ \frac{DS_{p}^{*}-DS_{p}^{n}}{\Delta t} & =\delta_{DS_{p}}\Delta DS_{p}^{*}. \end{array}$$

After obtaining S*, $S_{p}^{*}$, ES*, and $DS_{p}^{*}$ by calculating the diffusion part, the reaction parts were calculated to obtain *S*^*n*+1^, $S_{p}^{n+1}$, *E*^*n*+1^, *D*^*n*+1^, *ES*^*n*+1^, and $DS_{p}^{n+1}$. Specifically, for the full model (Eq 14), the following reaction equations were used.

$$\begin{array}{cc} \frac{S^{n+1}-S^{*}}{\Delta t} & =-k_{fe}S^{*}E^{n}+k_{be}ES^{*}+k_{d}{DS_{p}}^{*},\\ \frac{S_{p}^{n+1}-S_{p}^{*}}{\Delta t} & =-k_{fd}S_{p}^{*}D^{n}+k_{bd}DS_{p}^{*}+k_{e}ES^{*},\\ \frac{E^{n+1}-E^{n}}{\Delta t} & =-k_{fe}S^{*}E^{n}+\left(k_{be}+k_{e}\right)ES^{*},\\ \frac{D^{n+1}-D^{n}}{\Delta t} & =-k_{fd}S_{p}^{*}D^{n}+\left(k_{bd}+k_{d}\right)DS_{p}^{*},\\ \frac{ES^{n+1}-ES^{*}}{\Delta t} & =k_{fe}E^{n}S^{*}-k_{be}ES^{*}-k_{e}ES^{*},\\ \frac{DS_{p}^{n+1}-DS_{p}^{*}}{\Delta t} & =k_{fd}D^{n}S_{p}^{*}-k_{bd}DS_{p}^{*}-k_{d}DS_{p}^{*}. \end{array}$$

In the sQSSA_p_ model (Eq 15), we defined $E_{T}^{*}=E^{n}+ES^{*}$, $D_{T}^{*}=D^{n}+DS_{p}^{*}$, $ES^{*}\left(S^{*}\right)=\frac{E_{T}^{*}S^{*}}{S^{*}+K_{ME}}$, and $DS_{p}^{*}\left(S_{p}^{*}\right)=\frac{D_{T}^{*}S_{p}^{*}}{S_{p}^{*}+K_{MD}}$. Then, we obtained $S^{n+1},S_{p}^{n+1}$ as follows:

$$\begin{array}{cc} \frac{S^{n+1}-S^{*}}{\Delta t} & =-k_{e}ES^{*}\left(S^{*}\right)+k_{d}DS_{p}^{*}\left(S_{p}^{*}\right),\\ \frac{S_{p}^{n+1}-S_{p}^{*}}{\Delta t} & =k_{e}ES^{*}\left(S^{*}\right)-k_{d}DS_{p}^{*}\left(S_{p}^{*}\right). \end{array}$$

For the tQSSA_p_ model (Eq 16), we defined $E_{T}^{*}=E^{n}+ES^{*}$, $D_{T}^{*}=D^{n}+DS_{p}^{*}$, ${\hat{S}}^{*}=S^{*}+ES^{*}$, ${\hat{S}}_{p}^{*}=S_{p}^{*}+DS_{p}^{*}$, $ES^{*}\left({\hat{S}}^{*}\right)=\frac{1}{2}[E_{T}^{*}+{\hat{S}}^{*}+K_{ME}-\sqrt{{\left(E_{T}^{*}+{\hat{S}}^{*}+K_{ME}\right)}^{2}-4E_{T}^{*}{\hat{S}}^{*}}]$, and $DS_{p}^{*}\left({\hat{S}}_{p}^{*}\right)=\frac{1}{2}[D_{T}^{*}+{\hat{S}}_{p}^{*}+K_{MD}-\sqrt{{\left(D_{T}^{*}+{\hat{S}}_{p}^{*}+K_{MD}\right)}^{2}-4D_{T}^{*}{\hat{S}}_{p}^{*}}]$. Then, we obtained ${\hat{S}}^{n+1},{\hat{S}}_{p}^{n+1}$ as follows:

$$\begin{array}{cc} \frac{{\hat{S}}^{n+1}-{\hat{S}}^{*}}{\Delta t} & =-k_{e}ES^{*}\left({\hat{S}}^{*}\right)+k_{d}DS_{p}^{*}\left({\hat{S}}_{p}^{*}\right), \end{array}$$ (17)

$$\begin{array}{cc} \frac{{\hat{S}}_{p}^{n+1}-{\hat{S}}_{p}^{*}}{\Delta t} & =k_{e}ES^{*}\left({\hat{S}}^{*}\right)-k_{d}DS_{p}^{*}\left({\hat{S}}_{p}^{*}\right). \end{array}$$ (18)

For simulations, we used *k*_*fe*_ = *k*_*fd*_ = 2.22*μM*^−1^ *s*^−1^, *k*_*be*_ = *k*_*bd*_ = 1.84*s*^−1^, *k*_*d*_ = *k*_*e*_ = 0.38*s*^−1^, *K*_*ME*_ = *K*_*MD*_ = 1*μM*, and $\delta_{S}=\delta_{S_{p}}=\delta_{ES}=\delta_{DS_{p}}=0.2\mu m^{2}/s$. For discretization in space and time for Figs 4B and 4C, we used *L* = 30*μm*, *T* = 1,100*s*, *N*_*x*_ = *N*_*y*_ = 30, *N*_*t*_ = 100,000, resulting in Δ*x* = Δ*y* = 1*μm*, Δ*t* = 0.011*s* and the diffusion coefficients $\delta_{S}=\delta_{S_{p}}=\delta_{ES}=\delta_{DS_{p}}=0.2\mu m^{2}/s$. For Fig 4D and 4E, we used *L* = 30*μm*, *T* = 8.33*s*, *N*_*x*_ = *N*_*y*_ = 50, *N*_*t*_ = 200,000, resulting in Δ*x* = Δ*y* = 0.6*μm*, Δ*t* = 0.00004*s*.

The computational codes for implementing all of the models used in this study can be found at https://github.com/Mathbiomed/PDE_QSSA.

## Supporting information

S1 FigThe sQSSA_p_ model becomes inaccurate in heterogeneous environments, as long as the diffusion is slower than the slow reactions.The spatial average concentration of the product ($\bar{P}$) of the full model and that obtained from the sQSSA_p_ and tQSSA_p_ models were compared under varying diffusion coefficients (D_*_). Specifically, D_*_ was varied across a wide range, from 2 ⋅ 10^−5^*μm*^2^/*s* to 2 ⋅ 10^4^*μm*^2^/*s*, leading to a change in diffusion time scale (*L*^2^/D_*_) from 4.5 ⋅ 10^7^*s* to 4.5 ⋅ 10^−2^*s*. When the *L*^2^/D_*_ is ∼100-fold larger than the time scale of the slow reaction ($k_{cat}^{-1}=0.31s$), the sQSSA_p_ model overestimates $\bar{P}$, compared to the full model and the tQSSA_p_ model. When the *L*^2^/D_*_ is similar to or shorter than the time scale of the slow reaction, the sQSSA_p_ model also provides accurate results compared to the full model and the tQSSA_p_ model because the PDE behaves similarly to the ODE due to homogenization via fast diffusion.(TIF)
