# Supplementary figures and images for: Beyond homogeneity: Assessing the validity of the Michaelis–Menten rate law in spatially heterogeneous environments
Source: PLoS Comput Biol. 2024 Jun 6;20(6):e1012205. doi: 10.1371/journal.pcbi.1012205 (PMC11185478; doi:10.1371/journal.pcbi.1012205)

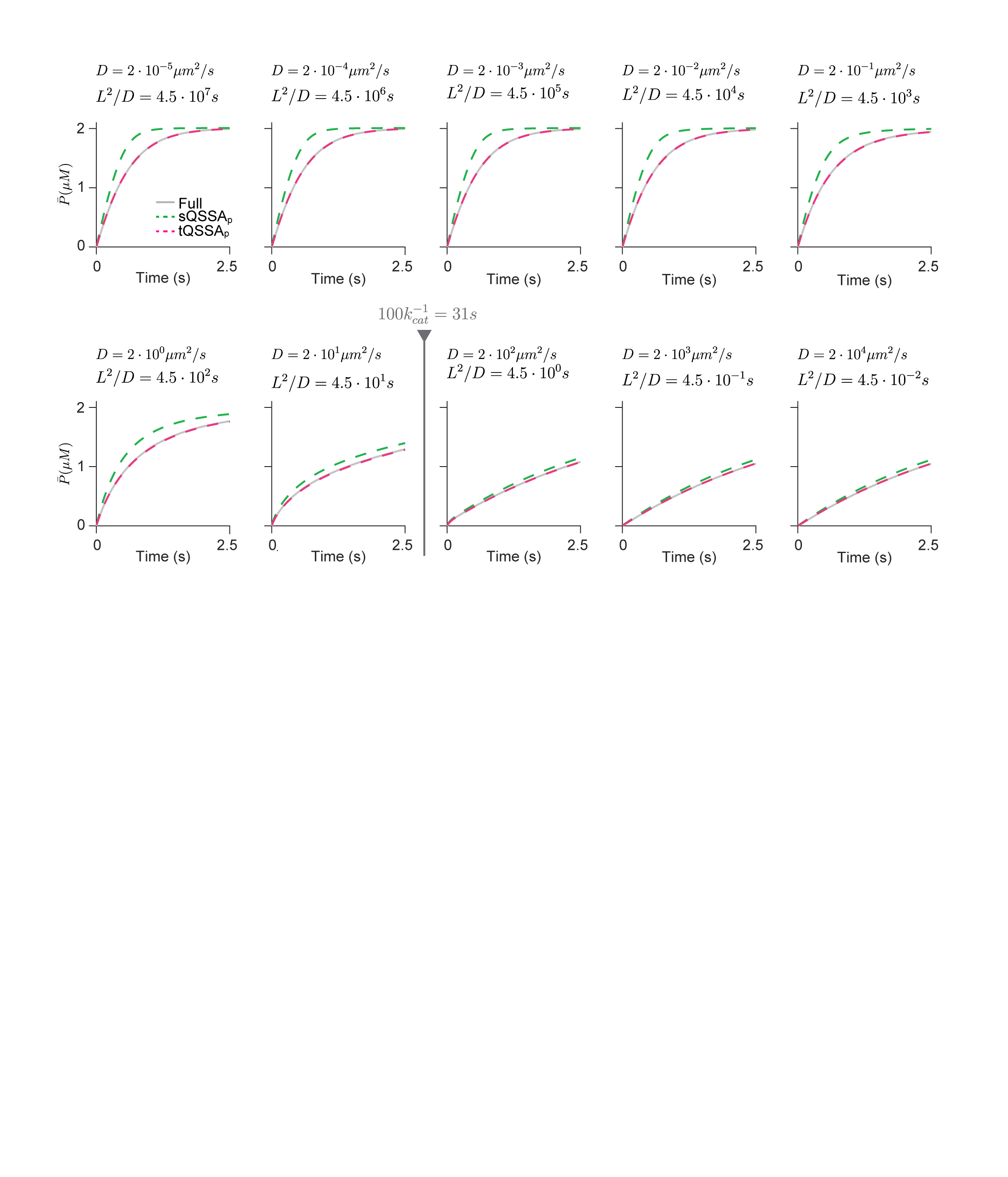

Supplement: S1 Fig — The spatial average concentration of the product (P¯) of the full model and that obtained from the sQSSAp and tQSSAp models were compared under varying diffusion coefficients (D*). Specifically, D* was varied across a wide range, from 2 ⋅ 10−5μm2/s to 2 ⋅ 104μm2/s, leading to a change in diffusion time scale (L2/D*) from 4.5 ⋅ 107s to 4.5 ⋅ 10−2s. When the L2/D* is ∼100-fold larger than the time scale of the slow reaction (kcat-1=0.31s), the sQSSAp model overestimates P¯, compared to the full model and the tQSSAp model. When the L2/D* is similar to or shorter than the time scale of the slow reaction, the sQSSAp model also provides accurate results compared to the full model and the tQSSAp model because the PDE behaves similarly to the ODE due to homogenization via fast diffusion. (TIF) [file pcbi.1012205.s001.tif]
